# Direct production of a genetically-encoded immobilized biodiesel catalyst

**DOI:** 10.1038/s41598-018-31213-y

**Published:** 2018-08-24

**Authors:** Bradley S. Heater, Marianne M. Lee, Michael K. Chan

**Affiliations:** School of Life Sciences and Center of Novel Biomaterials, The Chinese University of Hong Kong, Hong Kong SAR, China

## Abstract

The use of immobilized enzymes as biocatalysts has great potential to improve the efficiency and environmental sustainability of many industrial processes. Here, we report a novel approach that allows for the direct production of a highly active immobilized lipase within the bacterium *Bacillus thuringiensis*. Cry3Aa-lipA crystals were generated by genetically fusing *Bacillus subtilis* lipase A to Cry3Aa, a protein that naturally forms crystals in the bacteria. The crystal framework significantly stabilized the lipase against denaturation in organic solvents and high temperatures, resulting in a highly efficient fusion crystal that could catalyze the conversion of triacylglycerols to fatty acid methyl ester biodiesel to near-completion over 10 cycles. The simplicity and robustness of the Cry-fusion crystal (CFC) immobilization system could make it an appealing platform for generating industrial biocatalysts for multiple bioprocesses.

## Introduction

Enzymatic biocatalysis serves as a green approach to manufacture fine chemicals, pharmaceuticals and biofuels^1^. Immobilized enzymes are generally used since immobilization can enhance enzyme stability and recyclability, allowing for the facile recovery of the catalyst^2,3^. A major factor limiting the use of immobilized enzyme catalysts, however, is their production cost - both in terms of their isolation and purification, as well as their immobilization^4^.

One emerging strategy to help overcome these issues is the direct production of immobilized enzymes in bacteria. Examples of such genetically-encoded enzyme immobilization approaches include the use of self-aggregating peptides^5–7^ and protein domains^8–10^. These *in vivo* approaches simplify production by circumventing tedious purification and combining production and immobilization into a single step^11^. One difficult challenge, however, has been to produce a genetically-encoded immobilized catalyst that is both highly active and recyclable – key features of any potentially viable system. Previous studies have only been able to achieve one or the other. For example, self-aggregating peptides have been used to produce highly active immobilized enzymes, but to date there are no reports demonstrating their recyclability^5–7^. Alternatively, Diener *et al*. described the use of a coiled-coil domain to directly produce recyclable enzyme aggregates, but these aggregates exhibited much lower enzyme activities due to diffusion limitations^8^. Thus, if a genetically-encoded immobilized biocatalyst could be generated with both high activity and recyclability, it would be an important milestone in the development of these direct immobilization technologies.

Previously, our group demonstrated for the first time that when reporter proteins such as luciferase were genetically fused to the crystal-forming protein Cry3Aa^12,13^ and expressed in *Bacillus thuringiensis* (*Bt*), the resultant Cry-fusion crystals (CFCs) remained functionally active^14^. The crystalline nature of some Cry protein crystals produced in *Bt* has been confirmed by powder diffraction^15^, and in the case of Cry3Aa, its structure determination^16^. Though we have not confirmed this for our CFCs, electron microscopy (EM) has shown that their morphology and uniformity are similar to native Cry3Aa crystals^14^. As such, we loosely use the term crystals to reflect the regular shape and size of our CFCs compared to typical inclusions produced in bacteria.

In light of the retained activity of the Cry3Aa-luciferase crystals, it seemed plausible that the Cry3Aa framework could be used to directly produce other immobilized enzymes *in vivo*, with the hope that these crystals would retain high activity and recyclability. We surmised that if these particles preserved the natural channels intrinsic to Cry3Aa crystals, diffusion limitations should be minimal. Other favorable features associated with Cry proteins and their crystals include their strong promoters, long mRNA lifespan, high yields, release upon autolysis, column-free purification, and insolubility near neutral pH^17–19^. Moreover, due to the compact nature of crystals, CFCs may offer a dense population of enzyme monomers yet provide a regular spatial separation essential for robust enzyme activity.

We chose to explore the application of this strategy for lipases, the most widely used enzymes in the pharmaceutical^20^, cosmetic^21^ and most recently, biodiesel industries^22–24^. For our model lipase, we selected *Bacillus subtilis* lipase A (lipA), a small well-characterized 19 kDa minimal α/β-hydrolase with a plethora of crystal structures available^25–27^. Despite its pertinent industrial characteristics, lipA has had limited success in large-scale production, presumably because it aggregates easily during expression, purification and storage^28,29^. Notably, if lipA could be produced as a solid particle *in vivo*, it would eliminate the loss due to aggregation, and thus validate the use of the CFC framework for direct industrial enzyme immobilization and usage.

## Results and Discussion

### Production and characterization of Cry3Aa-lipA crystals

**Figure 1:**
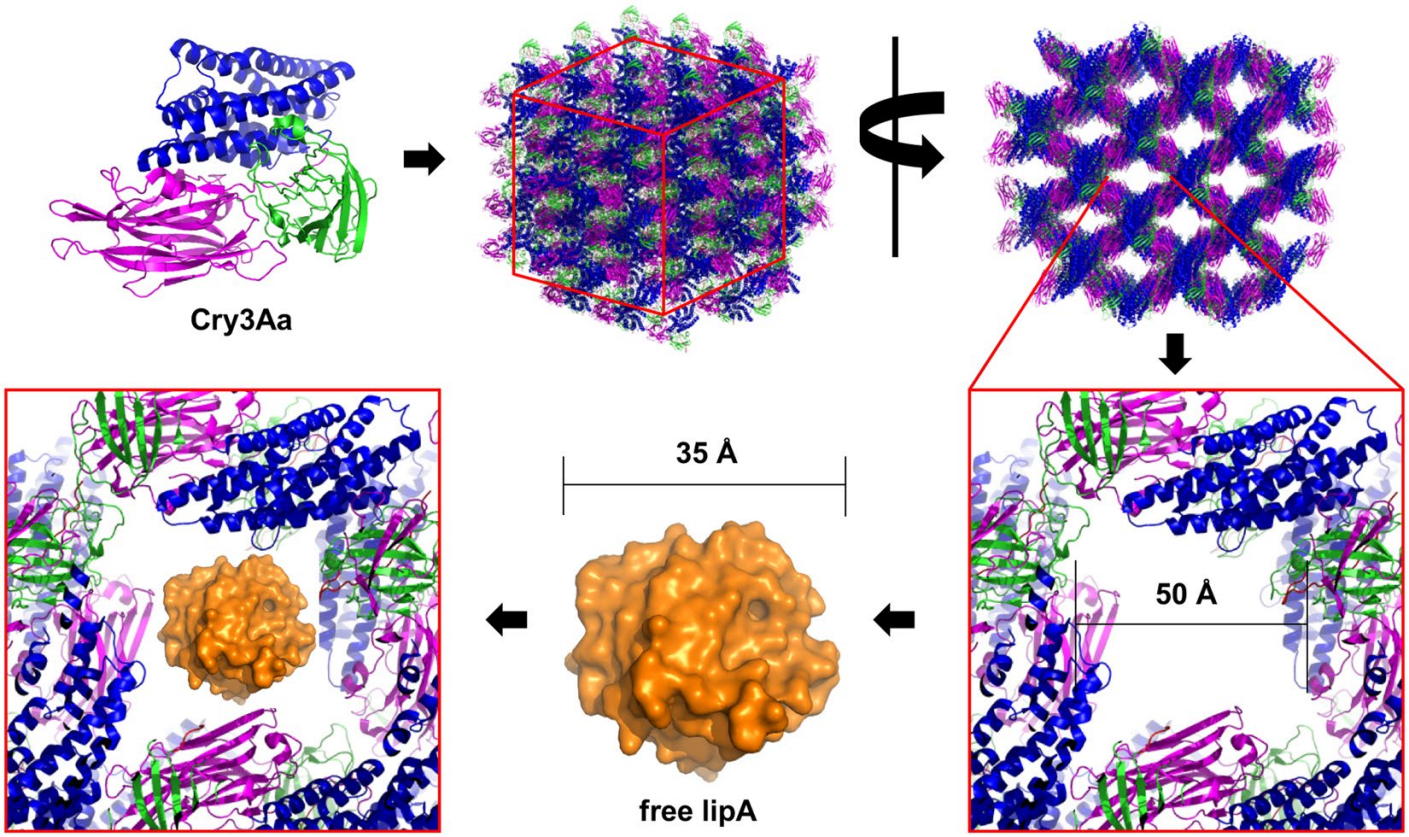
Schematic illustrating the design strategy used for optimization of Cry3Aa-lipA activity. Cry3Aa monomers form a tightly ordered crystal with ~50 Å by 50 Å channels^30^ that could easily accommodate free lipA^27^ (~35 Å).

Two Cry3Aa lipase fusion constructs were prepared and their resulting crystals tested for catalytic activity. One construct, Cry3Aa-lipA, was generated by directly fusing lipA to the C-terminus of Cry3Aa with the notion that immobilization was sufficient to generate a suitable catalyst^14^. The second construct, Cry3Aa*-lipA, was generated by deleting 19 amino acids from the C-terminus of Cry3Aa and incorporating a flexible GGGS linker between the modified Cry3Aa protein and lipA enzyme. The design of this second construct was based on our analysis of the existing Cry3Aa crystal structure (PDB ID: 1DLC)^30^ which suggested that this modification might better orient the fused lipA domains into the large channels present in Cry3Aa crystals (Fig. 1 and Supplementary Fig. 1), thereby providing them with greater substrate accessibility and increased conformational flexibility.

**Figure 2:**
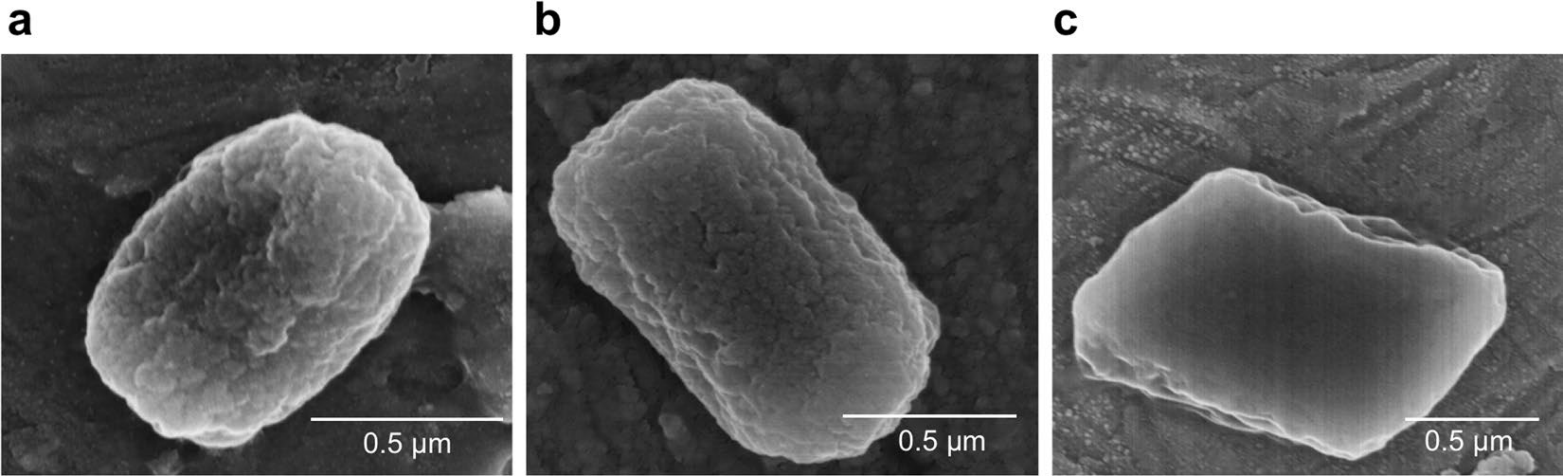
Single crystal comparison of Cry3Aa and Cry3Aa-lipA fusion crystals. (**a**) SEM of a purified Cry3Aa-lipA crystal, and (**b**) purified Cry3Aa*-lipA crystal at 60,000× magnification. (**c**) SEM of a purified Cry3Aa crystal at 45,000× magnification.

**Figure 3:**
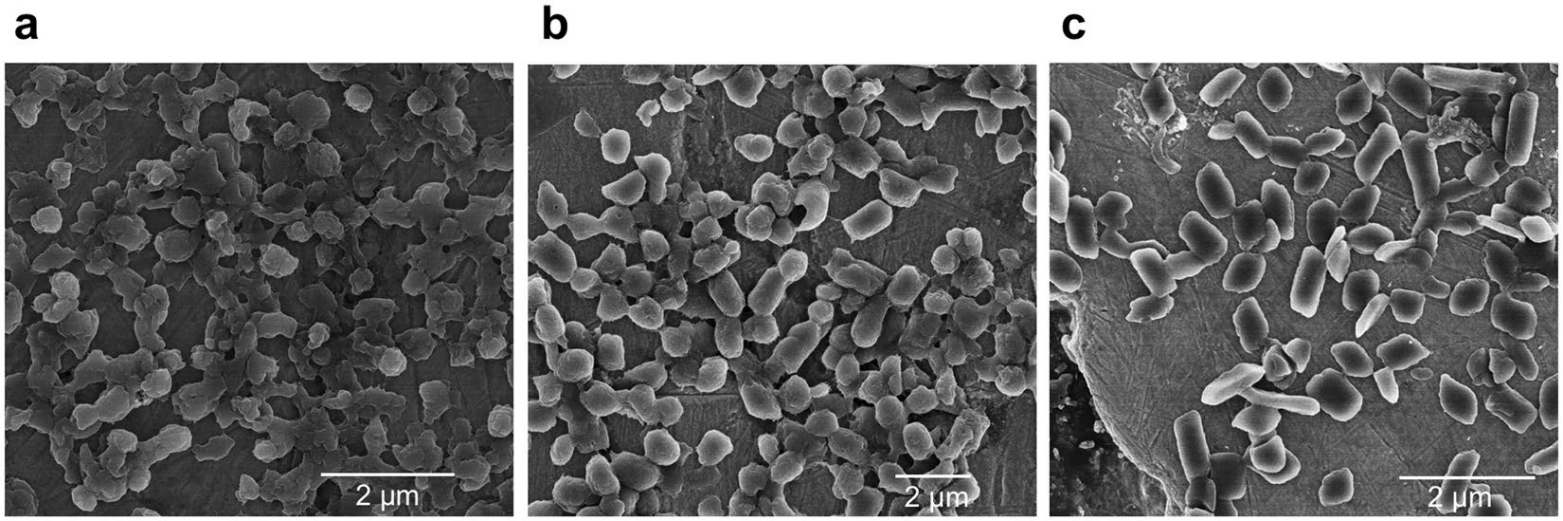
Comparison of Cry3Aa*-lipA particles produced in *Bt* and *E. coil*. (**a**) SEM of Cry3Aa*-lipA particles produced in *E. coli* at 11,000× magnification. (**b**) SEM of Cry3Aa*-lipA particles produced in *Bt* at 6,000× magnification. (**c**) SEM of Cry3Aa particles produced in *Bt* at 2,500× magnification.

Subsequent production of Cry3Aa-lipA and Cry3Aa*-lipA in *Bt* and isolation by ultracentrifugation yielded highly pure particles as demonstrated by SDS-PAGE gel electrophoresis (Supplementary Fig. 2). The presence of both Cry3Aa and lipA was verified by MALDI-TOF and LC-FTMS mass spectrometry (Supplementary Figs 3 and 4). Scanning electron microscopy (SEM) analysis of Cry3Aa-lipA and Cry3Aa*-lipA crystals revealed the particles to be mostly rod shaped (0.5 × 1.0 μm) with similar morphologies to native Cry3Aa crystals (Fig. 2). *E. coli* was also tested as a host for Cry3Aa*-lipA expression, but the particles generated were amorphous and significantly more heterogeneous in shape and size (Fig. 3a) than the Cry3Aa*-lipA and Cry3Aa particles produced in *Bt* (Fig. 3b,c).

Cry3Aa-lipA and Cry3Aa*-lipA production in *Bt* was extremely simple, requiring no column purification, and was achieved in high yield (116–122 mg/L protein crystals, which corresponds to 24.5 mg and 26.3 mg lipA, respectively) (Table 1). For comparison, free lipA was produced as a His-tagged fusion in *E. coli* and purified to near homogeneity. Due to precipitation during purification and dialysis, however, the final yield of free lipA was only 5.5 mg/L. Thus, production of lipA as a fusion with Cry3Aa in *Bt* resulted in a 4.4- to 4.8-fold improvement in yield compared to the free enzyme in *E. coli*. Subsequently, two other monomeric hydrolytic enzymes, an esterase (53 kDa) and a deformylase (19 kDa), were shown to be suitable for direct production as immobilized catalysts in high yields (Supplementary Fig. 5 and Table 2).

**Table 1:** Yield and specific activity of lipA constructs.

Construct	Yield (mg crystal/L)	Yield (mg lipA/L)	Activity (U/mg lipA)^a^	Activity retention (% free lipA)
free lipA	—	5.50	44.3 ± 2.8	100 ± 6.3
Cry3Aa-lipA	116	24.5	37.1 ± 2.4	83.7 ± 5.5
Cry3Aa*-lipA	122	26.3	98.1 ± 15	221 ± 33

^a^1 unit is the amount of enzyme required to produce 1 μmol pNP per min at 25 °C. Activity measurements were performed in triplicate, and the averages are shown with the standard deviation of the mean.

**Table 2:** Yield and specific activity of other Cry3Aa-fusion constructs.

Construct	Yield (mg crystals/L)	Yield (mg enzyme/L)	Fold increase in yield (crystal/free enzyme)	Activity (U/mg enzyme)	Activity retention (% free enzyme)
Cry3Aa-pnbA	106	44.5	1.21	^*a*^16.3 ± 0.50	101 ± 3.1
Cry3Aa-PDF	105	21.8	12.7	^*b*^28.2 ± 4.9	48.2 ± 8.4

^a^For pnbA,1 unit is the amount of enzyme required to produce 1 μmol pNP per min at 25 °C. ^*b*^For PDF, 1 unit is the amount of enzyme required to produce 1 μmol formate per h at 25 °C. Activity measurements were performed in triplicate, and the averages are shown with the standard deviation of the mean.

### Kinetic analyses

The hydrolytic activities of the two constructs were evaluated using the substrate *p*-nitrophenyl acetate (pNPA). Cry3Aa-lipA crystal and free lipA specific activities were comparable (37.1 U/mg and 44.3 U/mg, respectively), while Cry3Aa*-lipA exhibited a significantly higher specific activity of 98.1 U/mg (Table 1). To shed light on the observed activity improvement of Cry3Aa*-lipA, the kinetic parameters of these constructs were determined (Table 3 and Supplementary Fig. 6)^31^. Enzymes immobilized within matrices generally have increased K_M_ values relative to the free enzyme, since diffusional barriers can increase the substrate concentration required to completely saturate the enzyme active sites^32^. However, the *K*_M_ values for all lipA constructs were similar, indicating that unlike other immobilization platforms, internal diffusion and mass transfer limitations are not an issue^33–35^. One important difference, however, was the significantly higher *k*_cat_ of Cry3Aa*-lipA compared to Cry3Aa-lipA. This resulted in Cry3Aa*-lipA having a 2- and 3-fold enhancement in catalytic efficiency (*k*_cat_/*K*_M_) compared to free lipA and Cry3Aa-lipA, respectively. Although not structurally validated, we speculate that this enhancement is likely due to – as per our design – better orientation of the lipA monomers in the Cry3Aa channels. A favorable orientation of lipA may have increased the percentage of properly folded lipase in the crystal or promoted new interactions between Cry3Aa and lipA that stabilized a more active conformation^36^.

**Table 3:** Kinetic parameters of lipA constructs.

Construct	*K*_M_ (mM)	*k*cat (×10^3^ min^−1^)	*k*cat/*K*_M_ (×10^3^ mM^−1^ min^−1^)
free lipA	1.39 ± 0.10	1.85 ± 0.16	1.32 ± 0.02
Cry3Aa-lipA	1.61 ± 0.31	1.35 ± 0.25	0.84 ± 0.01
Cry3Aa*-lipA	1.19 ± 0.20	3.10 ± 0.34	2.61 ± 0.16

The values for *K*_M_ and *k*_cat_ were derived from the corresponding Lineweaver–Burke plots. Experiments were performed in triplicate, and the averages are shown with the standard deviation of the mean.

### Impact of CFC architecture on lipA stability

Owing to the improved activity of Cry3Aa*-lipA compared to Cry3Aa-lipA, further studies focused on this construct. We proceeded to verify the stability of the immobilized catalyst in solution – a key feature to its recyclability. Most immobilization strategies involve the use of bifunctional crosslinking reagents to link the enzyme molecules to either another enzyme molecule or a solid support. The required use of either of these agents is less attractive since they add to the cost of the immobilized catalyst and can lead to reductions in enzyme activity^37^.

**Figure 4:**
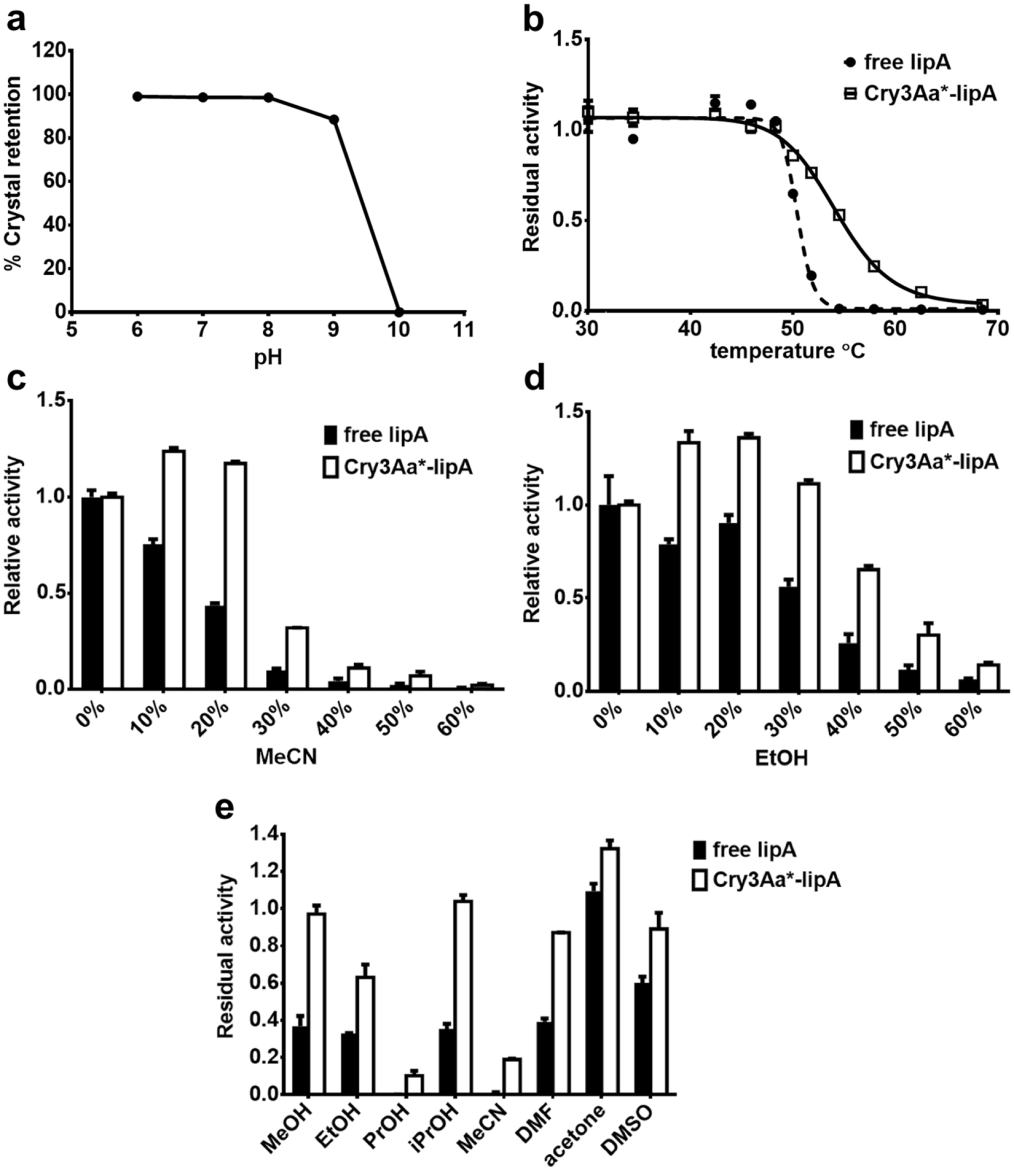
Stability of Cry3Aa*-lipA crystals. (**a**) Crystal retention of Cry3Aa*-lipA in various pH buffers at 2,000 rpm for 24 h. Values were normalized to the amount of protein solubilized at pH 10.0. (**b**) Thermal stability of free lipA and Cry3Aa*-lipA. Reactions were heated for 1 h at various temperatures prior to measuring residual activity, and then normalized to the residual activity at 30 °C. (**c**) Activity of free lipA and Cry3Aa*-lipA in increasing concentrations of MeCN and (**d**) EtOH. (**e**) 24 h stability of free lipA and Cry3Aa*-lipA in 50% v/v of MeOH, EtOH, PrOH, isopropyl alcohol (iPrOH), MeCN, dimethylformamide (DMF), acetone and dimethylsulfoxide (DMSO). All measurements were performed in triplicate. The error bars show the standard deviation of the mean. Error bars are present in Fig. 4a but the standard deviations are less than 1% so the error bars are difficult to see.

For Cry3Aa crystals, one of their key features is that they are naturally insoluble under the conditions used for most enzymatic reactions^18^. We reasoned that if the CFC-immobilized enzymes shared similar insolubility properties with their Cry3Aa parent, it might be possible to use them as catalysts without the need of any bifunctional agents. To evaluate this possibility, Cry3Aa*-lipA crystals were incubated with vigorous shaking in various pH buffers for 24 h, and the amount of solubilized protein was determined. The Cry3Aa*-lipA crystals were found to be stable to solubilization between pHs 6.0–8.0, with leakage of enzyme monomers only discernable at more alkaline pHs (Fig. 4a). Thus, crosslinking of Cry3Aa*-lipA is not needed to prevent crystal dissolution in a pH range between 6.0 and 8.0.

One important feature of any immobilization support for an industrial enzyme is its effect on enzyme stability against temperature and organic solvents. The impact of the Cry3Aa framework on the thermal stability of lipA was evaluated by heating Cry3Aa*-lipA crystals and free lipA in buffer for 1 h at various temperatures and measuring their residual activities. As displayed in Fig. 4b, Cry3Aa*-lipA crystals exhibited a T_50_ of 54.19 °C ± 0.23 compared to free lipA which had a T_50_ of 50.41 °C ± 0.11, suggesting that the Cry3Aa framework stabilizes lipA, thus making it well-suited for industrial processes. Cry3Aa-lipA (T_50_ 52.95 °C ± 0.25) was also more thermostable than free lipA (Supplementary Fig. 7), indicating that the increase in stability was not solely due to the C-terminal truncation of Cry3Aa, but rather by a different mechanism such as preventing irreversible aggregation of lipA monomers^28^. The slightly higher T_50_ of Cry3Aa*-lipA supports this model, since caging lipA within the channel might help prevent self-aggregation of lipA monomers and subsequent deactivation during heating.

The impact of the Cry3Aa architecture on lipA’s resistance to organic solvents was assessed by incubating Cry3Aa*-lipA crystals and free lipA in eight different aqueous organic solvents at 50% v/v for 24 h, and for acetonitrile (MeCN) and ethanol (EtOH), the activity was examined under a range of solvent concentrations (Fig. 4c–e). Cry3Aa*-lipA crystals were more active than free lipA in increasing concentrations of MeCN and EtOH (Fig. 4c,d), and maintained higher stability in the presence of all eight organic solvents tested (Fig. 4e). The most important finding from these solvent stability studies was that Cry3Aa*-lipA crystals retained nearly 100% activity after exposure to 50% v/v methanol (MeOH), suggesting that they might be suitable for transesterification reactions. As such, we chose to explore the application of Cry3Aa*-lipA crystals for the transesterification of coconut oil to fatty acid methyl ester (FAME) biodiesel, given the growing interest in green energy.

### Biodiesel production by Cry3Aa*-lipA crystals

**Figure 5:**
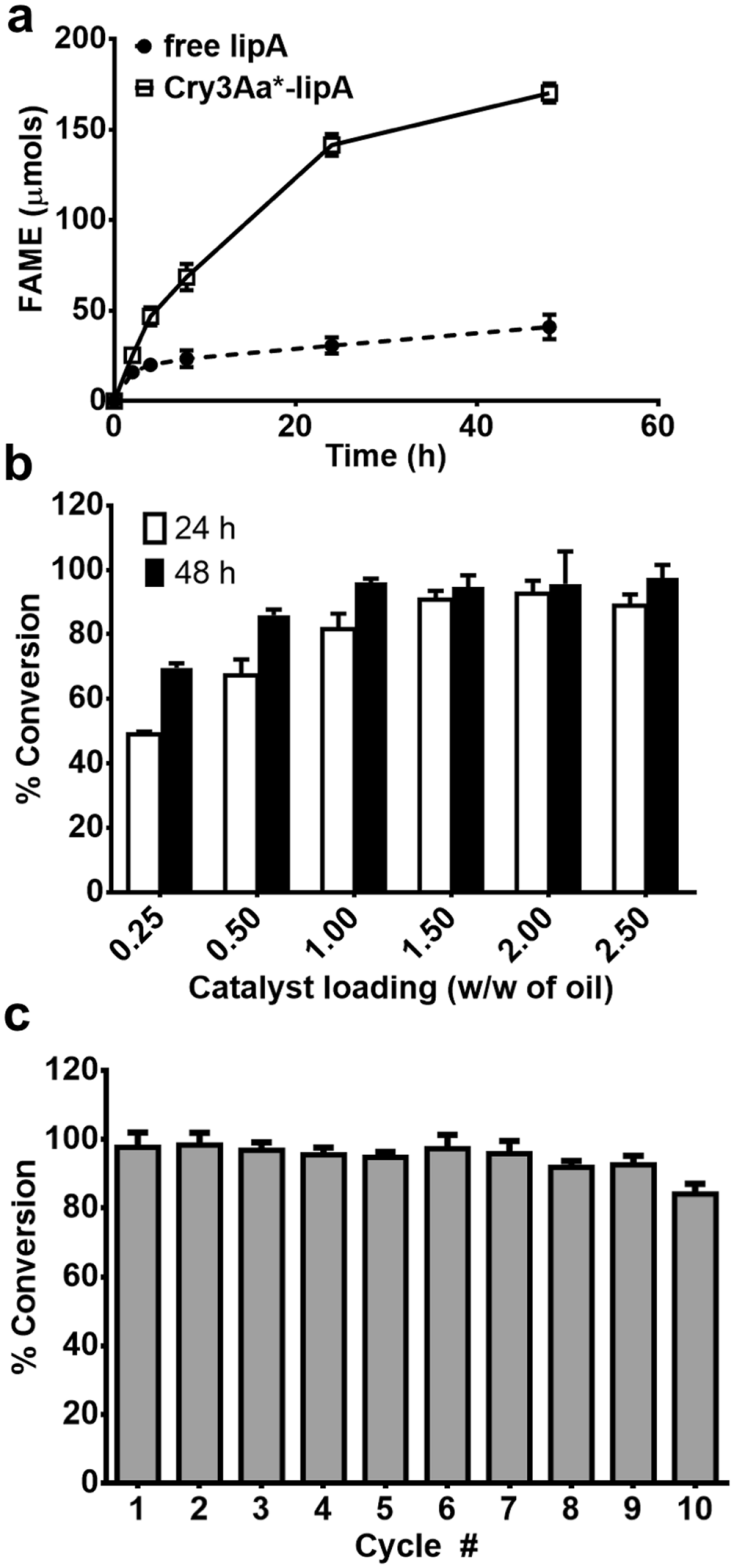
Biodiesel production by Cry3Aa*-lipA crystals. (**a**) A time-point study of the transesterification reaction of coconut oil and MeOH by free lipA and Cry3Aa*-lipA. At 2, 4, 8, 24, and 48 h samples were taken and analyzed by gas chromatography (GC). (**b**) Yield of coconut oil conversion to FAME biodiesel as a function of catalyst loading after 24 h and 48 h. (**c**) Recyclability of Cry3Aa*-lipA during the production of biodiesel from coconut oil using 2.5% (w/w) catalyst. All experiments were performed in triplicate. The error bars show the standard deviation of the mean.

FAME production by Cry3Aa*-lipA crystals was initially monitored under substrate saturating conditions. As shown in Fig. 5a, the initial rate of Cry3Aa*-lipA crystals was comparable to free lipA, though the rate diverged significantly after that. While Cry3Aa*-lipA continued to convert coconut oil to FAME biodiesel almost linearly for 24 h, free lipA activity plateaued after 2 h. This major difference is likely due to the much lower MeOH stability of free lipA compared to Cry3Aa*-lipA (Fig. 4e). With further optimization, 1.0% (w/w of oil) Cry3Aa*-lipA catalyst was found to be the minimum needed to reach a high conversion (96%) using a one-shot addition of MeOH. At 2.5% (w/w of oil) catalyst, however, a slightly higher conversion of 98% could be achieved (Fig. 5b). Recyclability was subsequently tested at 2.5% catalyst, and 93% conversion was maintained even after 9 reaction cycles (Fig. 5c), corresponding to a total turnover number of 49,713. This data demonstrates the robustness of the Cry3Aa*-lipA catalyst and its suitability for industrial transesterification processes. Notably, the 2.5% catalyst loading used in our study is significantly lower (7- to 18-fold) than two recently developed immobilized lipase constructs based on covalent immobilization^38^ and encapsulation^39^ thus highlighting the high enzyme packing capacity of our system.

### Summary

Cry proteins have been the subject of intense research due to their natural insecticidal properties. Different chimeras of *Bt* crystal-forming toxins have been generated in *Bt* with the aim of enhancing their insecticidal and larvicidal activities^40–44^. Our group, however, was the first to show the feasibility of producing functional protein fusion crystals and demonstrate their potential for *in vitro* and *in vivo* biological applications^14^. Notably, these previous Cry3Aa fusion particles displayed similar morphology as the Cry3Aa crystals based on EM data^14^. We have advanced our development of the CFC platform to the direct production of pure and active immobilized enzymes that can be used to catalyze specific reactions. We demonstrate that fusion of lipA to the crystal-forming protein Cry3Aa generates a promising catalyst for biodiesel production. The resulting Cry3Aa*-lipA crystals exhibit enhanced activity and stability, and more importantly, can be reused multiple times at low catalyst loadings and with high conversion efficiencies. We believe that the simplicity and uniqueness of our CFC technology could make it an elegant and cost-effective approach to produce other highly active and stable industrial biocatalysts.

## Materials and Methods

### Construction of expression plasmids

To construct the Cry3Aa fusion plasmids, the *lipA* and *pnbA* genes were amplified by PCR from *B. subtilis* 168 genomic DNA using Kapa HiFi (Kapa Biosystems) following the manufacturer’s instructions. The *pdf* gene from *Borrelia burgdorferi* was codon optimized for *Bacillus subtilis* expression and synthesized by Thermo-Scientific. All three genes were cloned downstream of the *cry3Aa* gene in the pHT315 vector^14^ using BamHI and KpnI sites via Gibson Assembly (NEB). This design results in constructs where the target enzyme is linked to the C-terminus of Cry3Aa by a GS linkage. Our studies have focused on C-terminal fusions because the N-terminal helix is known to undergo partial processing in the *Bt* cell^30^. Cry3Aa*-lipA was produced by amplifying the *cry3Aa* (1–625) gene and cloned upstream of the *lipA* gene using XhoI and BamHI sites in the pHT315 vector. A sequence encoding Gly-Gly-Gly was engineered in the reverse primer upstream of the BamHI site to incorporate a Gly-Gly-Gly-Gly-Ser flexible linker between Cry3Aa* and lipA. The plasmid for Cry3Aa*-lipA production in *E. coli* was constructed by amplifying the Cry3Aa*-lipA gene and cloning it into the NcoI and XhoI restriction sites in the pET28b vector via Gibson Assembly. To construct the free enzyme expression systems, the *lipA* and *pdf* genes were amplified by Kapa HiFi and cloned into the pET28b vector directly downstream of the gene encoding an N-terminal His-tag using the NdeI and XhoI restriction sites via Gibson Assembly. The *pnbA* gene was cloned downstream of SUMO using the Champion pET SUMO expression system (Invitrogen). All positive clones were verified by sequencing (BGI).

### Expression and purification of Cry3Aa-fusions and wild-type enzymes

All *Bt* Cry3Aa fusions were transformed, expressed and purified as previously described^14^. SDS-PAGE samples were prepared by solubilizing 30 μg crystals in 5X SDS dye and boiling for 10 min.

For Cry3Aa-fusion production in *E. coli* the pET28b Cry3Aa*-lipA plasmid was transformed into BL21 (DE3), and transformed colonies were inoculated into 500 mL Terrific Broth (IBI Scientific) supplemented with 50 μg/mL kanamycin (GoldBio). At OD 0.8, cells were induced with 1.0 mM isopropyl β-D-1-thiogalactopyranoside (IPTG, IBI Scientific) and incubated for 24 h at 30 °C, 220 rpm. The inclusions were purified as previously described^45^.

All wild-type enzymes were expressed in *E. coli* BL21 (DE3). Briefly, transformed colonies were inoculated into 500 mL LB (IBI Scientific) or 2x YT medium (Lab M) supplemented with 50 μg/mL kanamycin. At OD 0.6–0.8, cells were induced with 0.5 mM IPTG and incubated overnight at 25 °C, 220 rpm. All cell pellets were lysed by sonication and the cell lysate was loaded onto nickel affinity resin (GE) and eluted using an imidazole gradient (0–1 M) to yield the target proteins. The purity of the protein in each of the elution fractions was analyzed by SDS-PAGE.

More specifically, free lipA was purified in (50 mM Tris-HCl, 300 mM NaCl, pH 8.0 buffer)^46^, 1 mM benzamidine hydrochloride (Benz-HCL, TCI Chemicals), and the elutions containing pure lipA protein were combined and dialyzed into 10 mM NaH_2_PO_4_ pH 7.0 buffer. The highest yield obtained for free lipA was 5.5 mg/L. After dialysis, the aggregates were removed by centrifugation and filtration and the protein was concentrated using a 10 kDa cut-off ultrafiltration unit (Satorius) and used in subsequent assays.

Free PDF enzyme was purified as previously described^47^ with minor changes. The nickel purification was performed in 25 mM Tris-HCl, 500 mM NaCl, pH 8.0 buffer, 1 mM Benz-HCL, 1 mM phenylmethylsulfonyl fluoride (PMSF, Cayman Chemical), and fractions containing pure PDF were combined and precipitated using saturated ammonium sulfate, reconstituted in 20 mM HEPES, 10 mM NaCl pH 7.0 and further purified by an SEC70 gel filtration column (BioRad). Pure fractions were combined and concentrated in a 10 kDa cut-off ultrafiltration unit and stored at −80 °C.

The Champion pET SUMO expression system was used to produce native pnbA protein, where upon cleavage of His-SUMO-pnbA by SUMO protease yields the native pnbA enzyme. Pure fractions were concentrated using a 30 kDa cut-off ultrafiltration unit and aliquots were stored at −80 °C.

Protein concentrations were determined using the Bradford standard assay. The Bradford reagent (BioRad) can completely dissolve the CFCs to obtain soluble protein.

### Scanning Electron Microscopy

All CFCs were resuspended in deionized water to a final concentration of 0.1 mg/mL, and then 2 μL of this solution was added to a copper stub and allowed to dry overnight. Samples were coated with Au using a Sputter Coater S150B (Edwards) prior to imaging in an SU8000 (Hitachi) at 5 kV and at a working distance of 8.1 mm to 8.4 mm.

### MALDI-TOF

SDS-PAGE gel slices of Cry3Aa-lipA or Cry3Aa*-lipA were destained with MeOH, dried in a speed vac and digested with trypsin (Promega) overnight at 30 °C. Peptides were extracted by sonication in 80% MeCN/2.5% trifluoracetic acid (TFA) solution for 10 min, and subsequently analyzed by MALDI-TOF (Bruker UltrafleXtreme). Mass data acquisitions were piloted by FlexControl software using automatic run, and the data was searched by ProteinScape software 3.0 using MASCOT (Matrix Sciences) as a search engine against the NCBI database or Swiss protein database.

### LC-FTMS

Samples were prepared similarly to the MALDI-TOF samples, with an additional desalting step using a C18 Zip tip (Millipore) prior to analysis. Samples were analyzed using a Dionex Ultimate 3000 2D Nanoflow LC system Apex Ultra 7.0 Hybrid Qh-FTMS (Bruker Daltonics). Peptide fragment sequences were analyzed using Data Analysis 4.0.

### Enzyme kinetics

The activities of all lipA and pnbA constructs were measured using pNPA (Sigma) as a substrate. A stock solution of 100 mM pNPA was prepared in 100% MeCN. 50 μL of 3 mM pNPA was incubated with 50 μL of enzyme (3–100 nM) in 10 mM NaH_2_PO_4_ pH 7.0 buffer at 25 °C and the absorbance was measured at 405 nm at 20 s intervals using a Tecan M1000. The linear portion of the graph was used to calculate the activity in μmols *p*-nitrophenol (pNP) produced (ε = 18,000 M^−1^cm^−1^) per min. All kinetic parameters of lipA constructs were determined using pNPA as a substrate since it is soluble in water^31^. Initial rates of hydrolysis of pNPA at various concentrations were ascertained in 0.1 M NaH_2_PO_4_ pH 7.0 buffer. The values for *K*_M_ and *k*_cat_ were derived from the corresponding Lineweaver–Burke plots.

The activities of the PDF constructs were assayed by a formate dehydrogenase coupled assay using the formylated tripeptide f-MAS (Santa Cruz) as the substrate^48,49^. Reactions were initiated by adding 20 μL of 10 mM f-MAS to 40 μL of enzyme (0.125–2 μM) and incubating for 30 min at 25 °C. The reactions were stopped by boiling the reaction mixtures for 5 min. 50 μL of this reaction was then added to a 50 μL mixture containing 0.2 U/mL formate dehydrogenase (Sigma) and 2 mM NAD^+^ (Sigma). Reaction mixtures were incubated overnight at 37 °C and the formation of NADH was monitored every 10 min until completion at 340 nm using a Tecan M1000. The deformylation rate in μmols MAS formed per min was determined by measuring the amount of NADH produced (ε = 6,220 M^−1^cm^−1^) in the coupled reaction.

Activity retention of CFCs was determined by comparing the U/mg free enzyme to the U/mg CFC after accounting for the amount of enzyme within the fusion. For example, 1 mg of Cry3Aa-pnbA (as determined by Bradford) would be comprised of 0.42 mg pnbA and 0.58 mg of Cry3Aa calculated based on their respective molecular weights. For lipA and pnbA, 1 unit is the amount of lipA or pnbA required to produce 1 μmol pNP per min at 25 °C using 1.5 mM pNPA substrate. For PDF, 1 unit is the amount of PDF required to produce 1 μmol formate per h at 25 °C using 3.33 mM f-MAS substrate. All measurements were performed in at least triplicate. The error bars show the standard deviation of the mean.

### pH Stability of CFCs

Cry3Aa*-lipA crystals (2 mg/mL) were incubated for 24 h with vigorous shaking at 2,000 rpm in a thermomixer (Eppendorf) in 50 mM buffers of different pHs: Bis-Tris pH 6.0, HEPES pH 7.0, Tris-HCl pH 8.0–9.0, CAPS pH 10.0. The insoluble material was separated by centrifugation and the protein amount in the supernatant was determined using the Bradford assay. Percent crystal retention values were calculated by dividing the amount of protein solubilized by the amount of protein solubilized at pH 10.0. All measurements were performed in triplicate. The error bars show the standard deviation of the mean.

### Thermal stability

1 μM of free lipA and 1 μM Cry3Aa*-lipA were incubated in 10 mM NaH_2_PO_4_ pH 7.0 buffer at various temperatures for 1 h, cooled to RT for 10 min, and then assayed for residual activity as described in the Enzyme kinetics section above. Data was fit to a sigmoidal curve using the KaleidaGraph 4 software (Synergy Software). All measurements were performed in triplicate. The error bars show the standard deviation of the mean. The T_50_ values and standard deviations were calculated using GraphPad Prism software. T_50_ is defined as the temperature at which 50% activity remains after 1 h incubation.

### Organic solvent stability

To determine the 24 h stability in organic solvents, 1 μM free lipA or 1 μM Cry3Aa*-lipA were incubated in 50% v/v of MeOH, EtOH, PrOH, iPrOH, MeCN, DMF, acetone or DMSO at 25 °C, 300 rpm for 24 h, and then diluted 10-fold to 5% v/v solvent and assayed for residual activity as described in the Enzyme kinetics section. Residual activity was normalized to its initial activity in 5% corresponding solvent. All measurements were performed in triplicate. The error bars show the standard deviation of the mean.

### Activity in aqueous MeCN and EtOH

Catalyst activity in aqueous MeCN or EtOH was determined by mixing 30 μL of 100 nM free lipA or 100 nM Cry3Aa*-lipA in 10 mM NaH_2_PO_4_ pH 7.0 buffer with 120 μL of 3 mM pNPA in 0–75% MeCN or EtOH (final solvent concentrations were 0–60%). The activity was assayed as described in the Enzyme kinetics section. All activities were normalized to the activity in 100% buffer. All measurements were performed in triplicate. The error bars show the standard deviation of the mean.

### Biodiesel production

Transesterification as a function of time was done using 0.05 mg free lipA and 0.23 mg of Cry3Aa*-lipA (equivalent mol for mol) in a mixture containing 390 mg of 100% virgin pressed coconut oil (Tropicana, Local market) and 69.6 μL MeOH (3:1 MeOH: oil molar ratio) in 117 μl 10 mM NaH_2_PO_4_ pH 7.0 buffer (30% water w/w of oil), at 30 °C, 2,000 rpm. At 2, 4, 8, 24, and 48 h, 50 μL aliquots were centrifuged, and the oil layer was collect for analysis by gas chromatography (GC).

Determination of conversion as a function of catalyst loading was performed using various amounts of catalyst (0.25%–2.5% w/w of oil), 130 mg coconut oil and 23.2 μL MeOH in 39 μl 0.1 M NaH_2_PO_4_ pH 7.0 buffer, at 2,000 rpm and 30 °C, and samples were collected at 24 and 48 h and analyzed by GC.

For the recyclability reaction, 2.5% (w/w) Cry3Aa*-lipA catalyst loading was used to evaluate its impact on FAME yields over multiple reaction cycles. Accordingly, 9.75 mg Cry3Aa*-lipA crystals was added to 390 mg coconut oil, and the mixture was briefly sonicated to attain homogeneity. Then, 69.6 μL MeOH and 117 μL of 0.1 M NaH_2_PO_4_ pH 7.0 buffer were added to the catalyst-oil mixture and incubated in a thermomixer at 30 °C, 2,000 rpm for 48 h. After each 48 h cycle, the reaction mixtures were centrifuged at high speed for 5 min and the oil layer was extracted and analyzed by GC. The insoluble crystals were washed twice with 1 mL hexane and collected by centrifugation and subsequently dried in a speed-vac for 10 min. They were then washed once with 1 mL of 0.1 M NaH_2_PO_4_ pH 7.0 buffer, centrifuged and collected before being dried in a speed-vac for 10 min. Fresh oil, MeOH and buffer were added to initiate the next cycle. All measurements were performed in triplicate. The error bars show the standard deviation of the mean.

### Quantification of Biodiesel

The extent of biodiesel formation was determined by mixing 5 μL of the oil layer with 0.5 mL of 0.5 mg/mL methyl heptadecanoate (internal standard) in hexane and analyzed by GC. 1 μL samples were injected at a 20:1 split ratio in an Agilent Technologies 7890B GC System equipped with a flame ionized detector (FID) using a Select Biodiesel FAME column (30 m × 0.32 mm × 0.25 μm, Agilent). The carrier gas used was helium at a flow rate of 1.5 mL/min. The oven temperature was kept at 200 °C for 3 min and then increased to 230 °C at 3 °C/min and held at 230 °C for 3 min. The temperature was subsequently raised to 250 °C at 10 °C/min and held at 250 °C for 6 min. The injector and detector temperatures were set at 260 °C and 300 °C respectively. The percent conversion was determined by comparing to a biodiesel sample prepared from coconut oil using a large excess of free *Burkholderia cepacia* lipase (Sigma) at a 5:1 MeOH: oil ratio for 48 h. A large excess of *B. cepacia* lipase has been shown to reach complete conversion using other feedstock oils^38,50^. Thin layer chromatography (TLC) was used to verify that all the coconut oil was converted to FAME by *B. cepacia* lipase (Supplementary Fig. 8). The method for TLC was performed as described previously^51^, but I_2_ vapor was used for the staining.

## Electronic supplementary material

Supplementary Information
