# Supplementary material for: Direct production of a genetically-encoded immobilized biodiesel catalyst
Source: Sci Rep. 2018 Aug 24;8:12783. doi: 10.1038/s41598-018-31213-y (PMC6109139; doi:10.1038/s41598-018-31213-y)
Supplement: Supplementary file 1 — Supplementary Information [file 41598_2018_31213_MOESM1_ESM.pdf]

# Supplementary Information

# Direct production of a genetically-encoded immobilized biodiesel catalyst

**Bradley S. Heater, Marianne M. Lee & Michael K. Chan***

School of Life Sciences and Center of Novel Biomaterials, The Chinese University of Hong Kong, Hong Kong SAR, China.

*Email: michaelkchan88@cuhk.edu.hk

# Supplementary Information

## Supplementary Figures

### Supplementary Figure 1

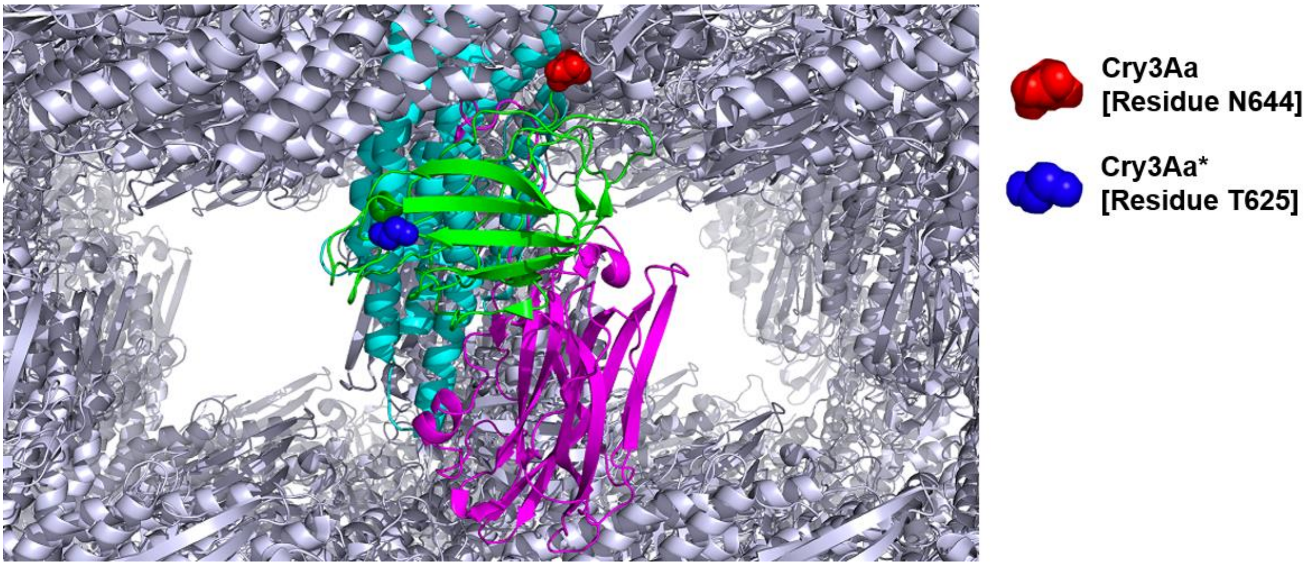


**Location of C-terminal amino acid of Cry3Aa and Cry3Aa* in the Cry3Aa crystal channel[30].** The C-terminal residue of Cry3Aa (residue N644 in red spheres) and Cry3Aa* (residue T625 in blue spheres) are shown for a single Cry3Aa monomer in the Cry3Aa crystal lattice. Since the C-terminus of Cry3Aa is more buried in the crystal, while that of Cry3Aa* is pointed towards the channel's center, fusion of lipA to Cry3Aa* could better position lipA in the solvent channel.

**Supplementary Figure 2**

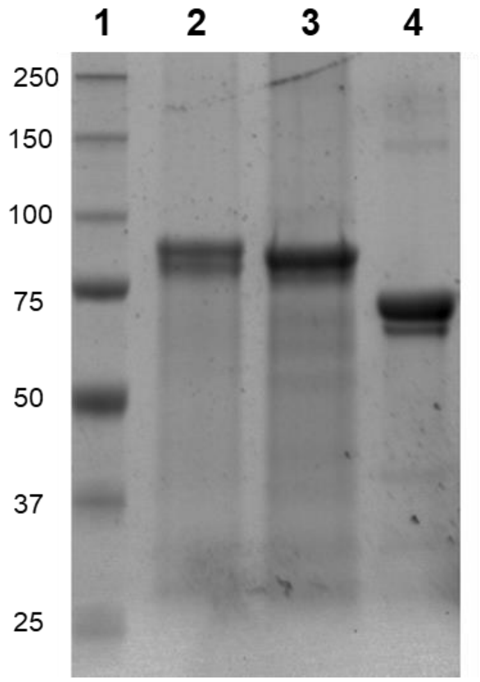


**SDS-PAGE of Cry3Aa lipA fusion crystals.** Lane (1) Molecular weight marker (kDa). Lane (2) Purified Cry3Aa-lipA crystals. Lane (3) Purified Cry3Aa*-lipA crystals. Lane (4) Purified Cry3Aa crystals. 4-5 μg crystals were solubilized in 5X SDS dye, boiled and loaded onto a 10% TGX Stain-Free gel (BioRad). The minor band just below each major band is due to partial processing of the Cry3Aa N-terminal helix in the *Bt* cell[30]. We have determined that N-terminal truncation of Cry3Aa-lipA does not impact its activity or stability (data not shown).

# Supplementary Information

## Supplementary Figure 3

**a**

| | |
|---|---|
| **Search Type** | Combined MS/MS - ProteinExtractor |
| **Search Result** | NCBInr All 100ppm_2017-08-18 15:35:48 |
| **Search Location** | /Prof. Michael Chan 2017/MSMS20170818Brad/MSMS20170818/A8/CombinedLIFT/ |

**Protein 1:** Chain A, Crystal Structure Of Insecticidal Delta-Endotoxin From Bacillus Thuringiensis At 2.5 Angstroms Resolution

| | | | |
|---|---|---|---|
| **Accession:** | gi\|157830835 | **Score:** | 405.90 |
| **Database:** | NCBInr | **MW [kDa]:** | 66.20 |
| **Seq. Coverage [%]:** | 12.50 % | **pI:** | 5.68 |
| | | **No. of Peptides:** | 6 |

| Cmpd. | No. of Cmpds. | m/z meas. | Δ m/z [ppm] | z | Rt [min] | Score | Site [%] | P | Range | Sequence | Modification | Type |
|---|---|---|---|---|---|---|---|---|---|---|---|---|
| | 1 | 1350.6372 | -4.81 | 1 | | 67.17 | | 0 | 108-118 | R.ELFSQAESHFR.N | | CID |
| | 1 | 1445.6362 | -5.65 | 1 | | 59.51 | | 0 | 196-207 | R.GSSYESWVNFNR.Y | | CID |
| | 1 | 1524.8284 | -6.32 | 1 | | 86.98 | | 0 | 242-255 | R.DVLTDPIVGVNNLR.G | | CID |
| | 1 | 1634.7726 | -5.06 | 1 | | 97.95 | | 0 | 256-269 | R.GYGTTFSNIENYIR.K | | CID |
| | 1 | 1325.7109 | -0.28 | 1 | | 40.41 | | 0 | 270-279 | R.KPHLFDYLHR.I | | CID |
| | 1 | 1141.6347 | 1.98 | 1 | | 53.88 | | 0 | 460-471 | K.LQSGASVVAGPR.F | | CID |

**Protein 2:** esterase, partial [Bacillus subtilis]

| | | | |
|---|---|---|---|
| **Accession:** | gi\|518542140 | **Score:** | 86.16 |
| **Database:** | NCBInr | **MW [kDa]:** | 16.70 |
| **Seq. Coverage [%]:** | 16.10 % | **pI:** | 9.90 |
| | | **No. of Peptides:** | 2 |

| Cmpd. | No. of Cmpds. | m/z meas. | Δ m/z [ppm] | z | Rt [min] | Score | Site [%] | P | Range | Sequence | Modification | Type |
|---|---|---|---|---|---|---|---|---|---|---|---|---|
| | 1 | 1392.6786 | -5.79 | 1 | | 32.00 | | 0 | 76-88 | K.TGTNYNNGPVLSR.F | | CID |
| | 1 | 1170.6578 | -1.00 | 1 | | 54.16 | | 0 | 127-138 | K.VANVVTLGGANR.L | | CID |

**b**

| | |
|---|---|
| **Search Type** | Combined MS/MS - ProteinExtractor |
| **Search Result** | NCBInr All 100ppm_2017-08-18 15:35:48 |
| **Search Location** | /Prof. Michael Chan 2017/MSMS20170818Brad/MSMS20170818/B8/CombinedLIFT/ |

**Protein 1:** Chain A, Crystal Structure Of Insecticidal Delta-Endotoxin From Bacillus Thuringiensis At 2.5 Angstroms Resolution

| | | | |
|---|---|---|---|
| **Accession:** | gi\|157830835 | **Score:** | 508.42 |
| **Database:** | NCBInr | **MW [kDa]:** | 66.20 |
| **Seq. Coverage [%]:** | 12.50 % | **pI:** | 5.68 |
| | | **No. of Peptides:** | 6 |

| Cmpd. | No. of Cmpds. | m/z meas. | Δ m/z [ppm] | z | Rt [min] | Score | Site [%] | P | Range | Sequence | Modification | Type |
|---|---|---|---|---|---|---|---|---|---|---|---|---|
| | 1 | 1350.6463 | 1.96 | 1 | | 72.29 | | 0 | 108-118 | R.ELFSQAESHFR.N | | CID |
| | 1 | 1445.6465 | 1.43 | 1 | | 61.86 | | 0 | 196-207 | R.GSSYESWVNFNR.Y | | CID |
| | 1 | 1524.8400 | 1.31 | 1 | | 103.37 | | 0 | 242-255 | R.DVLTDPIVGVNNLR.G | | CID |
| | 1 | 1634.7878 | 4.20 | 1 | | 112.00 | | 0 | 256-269 | R.GYGTTFSNIENYIR.K | | CID |
| | 1 | 1325.7102 | -0.82 | 1 | | 68.46 | | 0 | 270-279 | R.KPHLFDYLHR.I | | CID |
| | 1 | 1141.6322 | -0.19 | 1 | | 90.44 | | 0 | 460-471 | K.LQSGASVVAGPR.F | | CID |

**Protein 2:** esterase, partial [Bacillus subtilis]

| | | | |
|---|---|---|---|
| **Accession:** | gi\|518542140 | **Score:** | 128.16 |
| **Database:** | NCBInr | **MW [kDa]:** | 16.70 |
| **Seq. Coverage [%]:** | 22.60 % | **pI:** | 9.90 |
| | | **No. of Peptides:** | 3 |

| Cmpd. | No. of Cmpds. | m/z meas. | Δ m/z [ppm] | z | Rt [min] | Score | Site [%] | P | Range | Sequence | Modification | Type |
|---|---|---|---|---|---|---|---|---|---|---|---|---|
| | 1 | 1182.5924 | 1.84 | 1 | | 36.34 | | 0 | 55-64 | K.SYLVSQGWSR.D | | CID |
| | 1 | 1392.6859 | -0.51 | 1 | | 39.94 | | 0 | 76-88 | K.TGTNYNNGPVLSR.F | | CID |
| | 1 | 1170.6589 | -0.06 | 1 | | 51.88 | | 0 | 127-138 | K.VANVVTLGGANR.L | | CID |

**Matrix-assisted laser ionization time-of-flight mass spectrometry (MALDI-TOF) analysis of Cry3Aa-lipA fusions.** (**a**) SDS-PAGE gel slices of Cry3Aa-lipA or (**b**) Cry3Aa*-lipA were digested with trypsin and peptides were extracted and subsequently analyzed by MALDI-TOF. Peptides from both Cry3Aa and esterase (lipA) were detected in the fusions.

## Supplementary Information

### Supplementary Figure 4

a

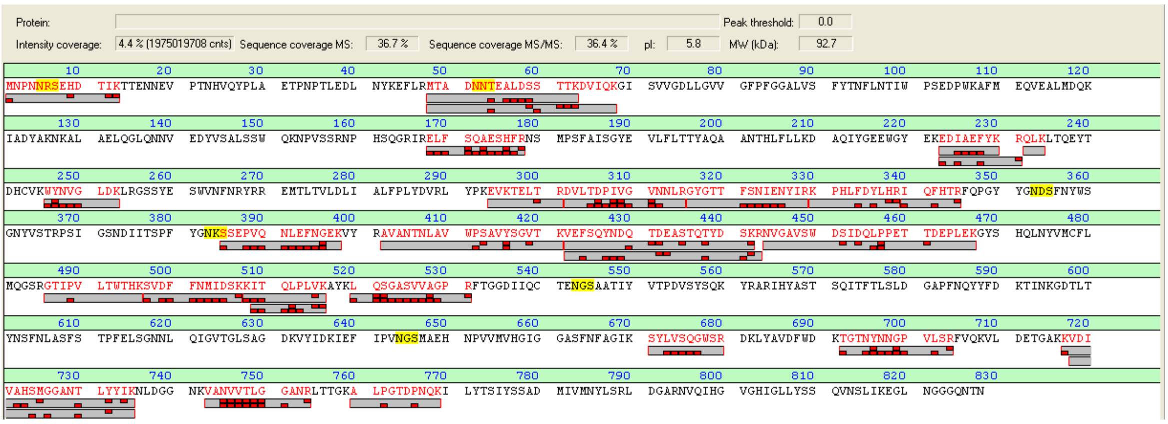

b

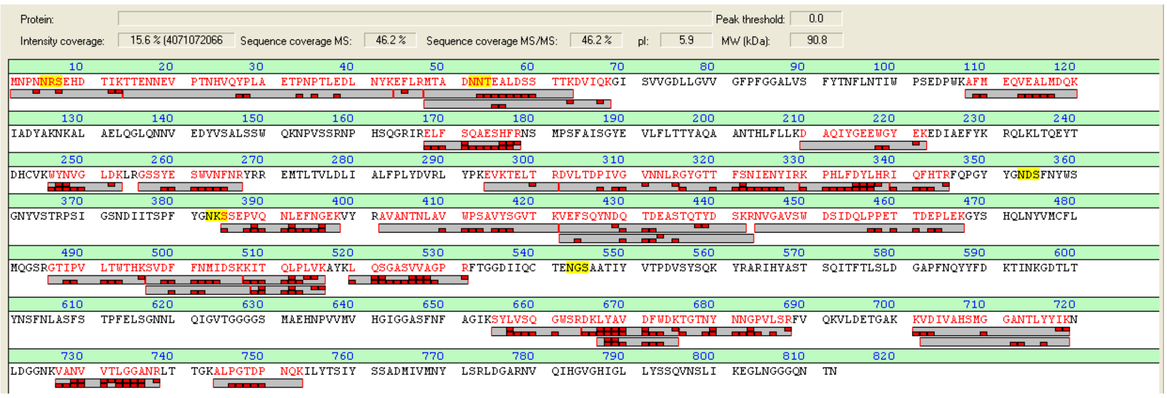

**Fourier Transform Mass Spectrometry (FTMS) analysis of Cry3Aa-lipA fusions.** (**a**) SDS-PAGE gel slices of Cry3Aa-lipA or (**b**) Cry3Aa*-lipA were digested with trypsin and peptides were extracted and subsequently analyzed by FTMS. Amino acids labeled red were detected as peptide fragments (gray box) and subsequently analyzed by MS/MS. Red boxes indicate detected amino acids by MS/MS. Peptides from Cry3Aa and lipA could be detected in both bands.

**Supplementary Figure 5**

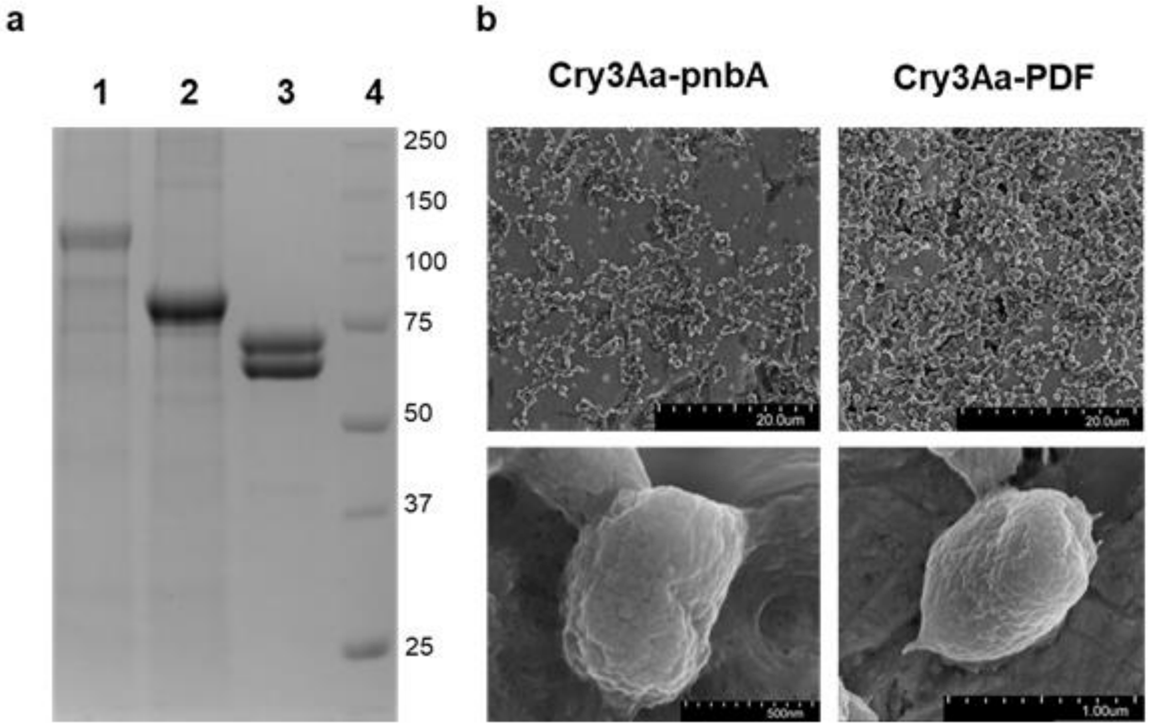


**Production of two other Cry3Aa-enzyme fusion constructs** *p*-Nitrobenzyl esterase from *Bacillus subtilis* 168 (pnbA, 53 kDa) and peptide deformylase from *Borrelia burgdorferi* (PDF, 19 kDa) were genetically fused to Cry3Aa and produced in *Bt*. (**a**) SDS-PAGE of Cry3Aa-enzyme fusion constructs. Lane (1) Purified Cry3Aa-pnbA crystals. Lane (2) Purified Cry3Aa-PDF crystals. (3) Purified Cry3Aa crystals. The occurrence of two major bands is due to partial processing of the Cry3Aa N-terminal helix in the *Bt* cell[30]. Lane (4) Molecular weight marker (kDa). Crystals were solubilized in 5X SDS dye, boiled and 4-5 μg was loaded onto a 10% TGX Stain-Free gel. (**b**) SEM of purified Cry3Aa-pnbA and Cry3Aa-PDF crystals at 2,500X and 60,000X magnification.

## Supplementary Figure 6

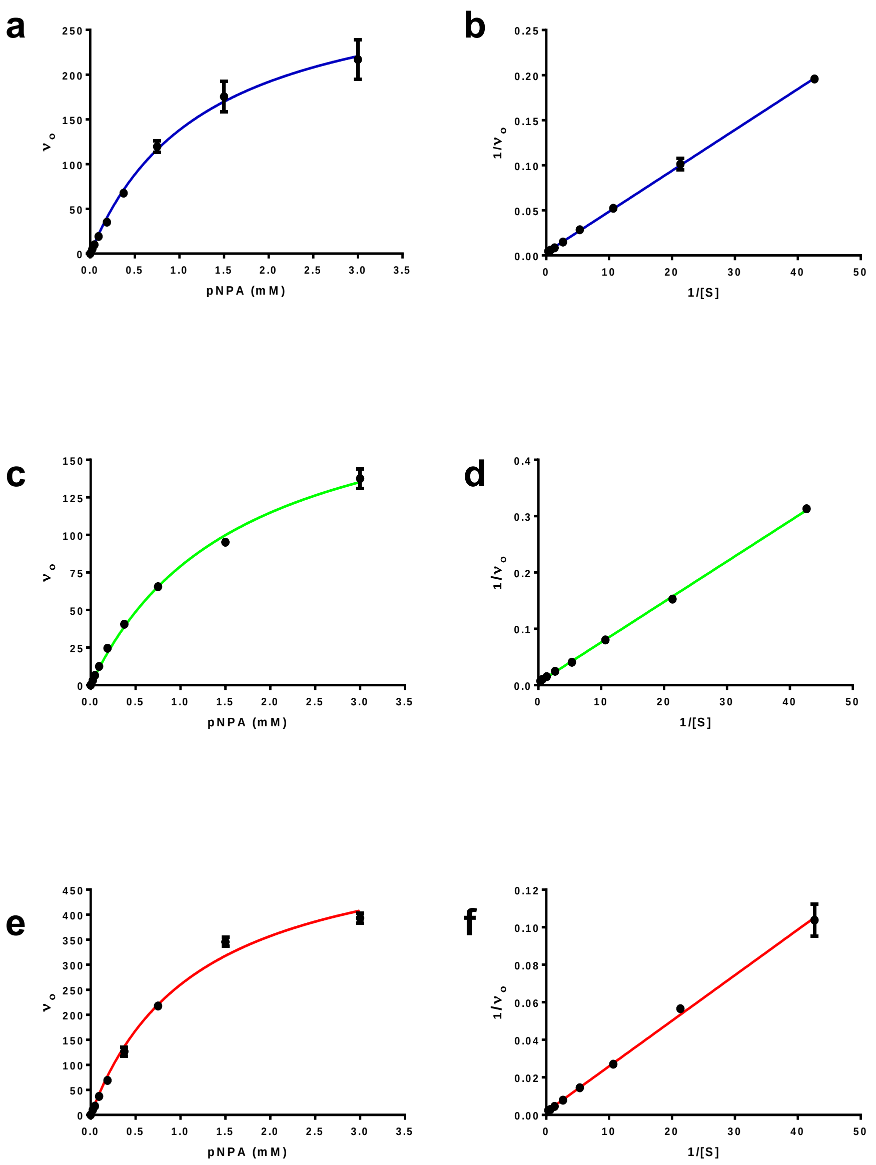


**Kinetics of lipA constructs.** (**a**) Plot of initial rate of free lipA in the presence of varying amounts of pNPA and (**b**) corresponding Lineweaver-Burk plot. (**c**) Plot of initial rate of Cry3Aa-lipA and (**d**) corresponding Lineweaver-Burk plot. (**e**) Plot of initial rate of Cry3Aa*-lipA and (**f**) corresponding Lineweaver-Burk plot. Rates are in nmols $L^{-1}$ $s^{-1}$.

**Supplementary Figure 7**

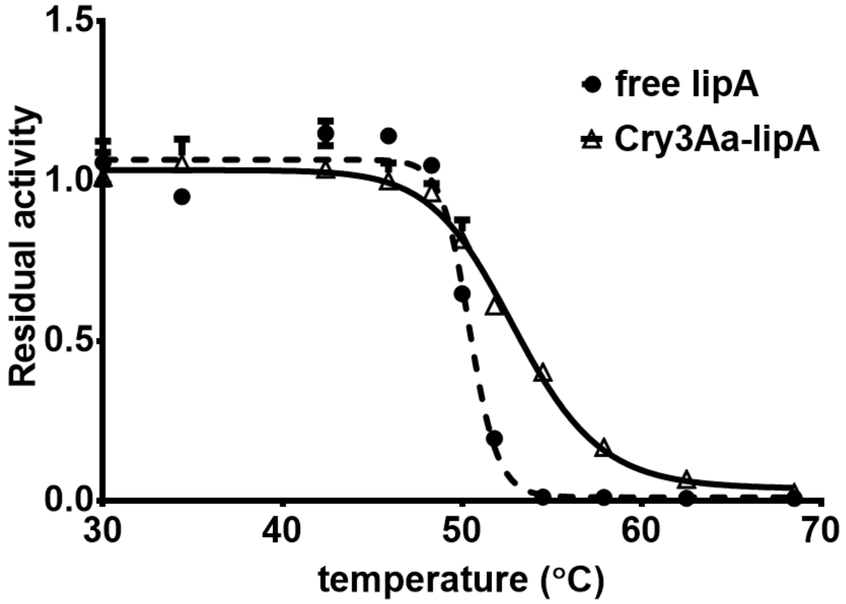


**Thermal stability of Cry3Aa-lipA and free lipA.** Free lipA and Cry3Aa-lipA were heated for 1 h at various temperatures and the residual activities were measured. Activities were normalized to the activity at 30°C. All measurements were performed in triplicate. The error bars show the standard deviation of the mean.

**Supplementary Figure 8**

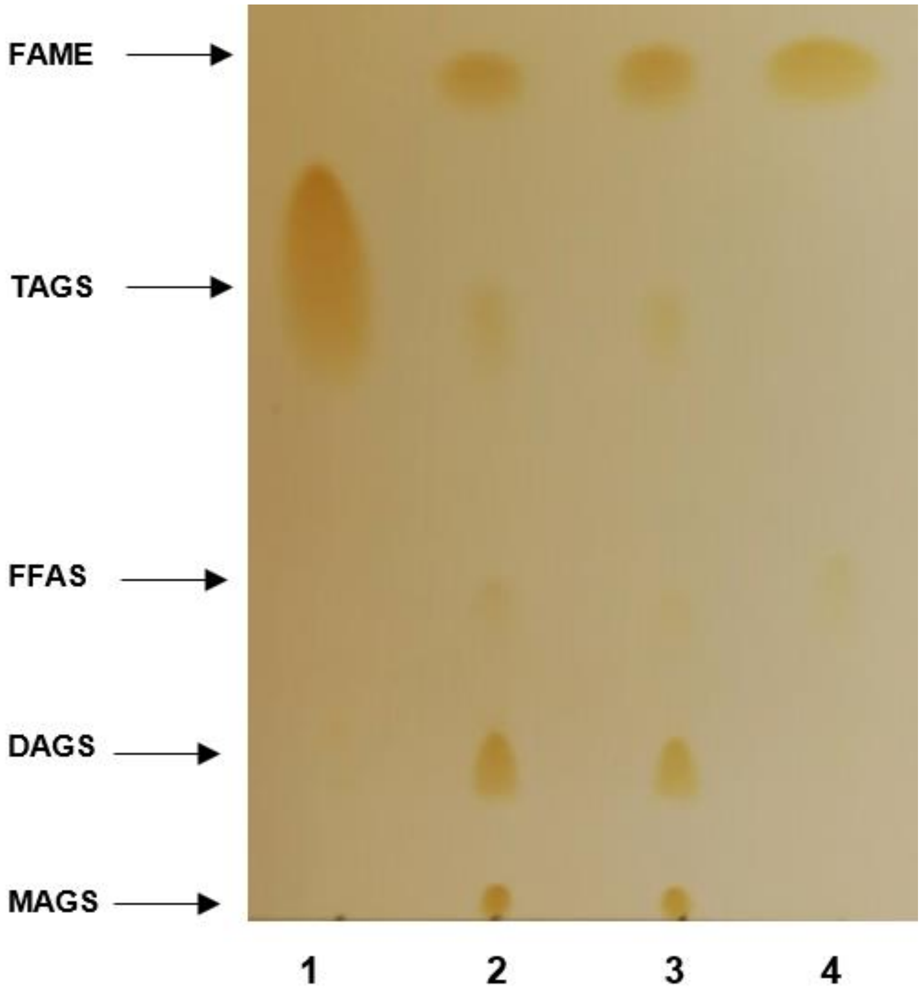


**TLC of FAME produced by *B. cepacia* lipase.** (**1**) Conversion of coconut oil to FAME after 0 min, (**2**) 5 min, (**3**) 1 h, and (**4**) 48 h. Fatty acid methyl esters (FAME), triacylglycerols (TAGS), free fatty acids (FFAs), diacylglycerols (DAGs) and monoacylglycerols (MAGs) are indicated with arrows.
